# Genetic and Epigenetic Characterization of a Discordant *KMT2A/AFF1*-Rearranged Infant Monozygotic Twin Pair

**DOI:** 10.3390/ijms22189740

**Published:** 2021-09-09

**Authors:** Alessia Russo, Clara Viberti, Katia Mareschi, Elisabetta Casalone, Simonetta Guarrera, Giovanni Birolo, Giovanni Cazzaniga, Lilia Corral, Luca Trentin, Giuseppe Basso, Franca Fagioli, Giuseppe Matullo

**Affiliations:** 1 Department of Medical Sciences, University of Turin, 10126 Turin, Italy; alessia.russo@unito.it (A.R.); clara.viberti@unito.it (C.V.); elisabetta.casalone@unito.it (E.C.); giovanni.birolo@unito.it (G.B.); 2 Stem Cell Transplantation and Cellular Therapy Laboratory, Paediatric Onco-Haematology, City of Health and Science of Turin, Regina Margherita Children’s Hospital, 10126 Turin, Italy; katia.mareschi@unito.it (K.M.); franca.fagioli@unito.it (F.F.); 3 Department of Public Health and Paediatrics, University of Turin, 10126 Turin, Italy; 4 IIGM-Italian Institute for Genomic Medicine, c/o IRCCS, 10060 Candiolo, Italy; simonetta.guarrera@iigm.it; 5 Candiolo Cancer Institute, FPO-IRCCS, 10060 Candiolo, Italy; 6 M.Tettamanti Research Center, Pediatric Clinic University of Milano-Bicocca, 20900 Monza, Italy; gianni.cazzaniga@asst-monza.it (G.C.); liliacorralabascal@gmail.com (L.C.); 7 Onco-Hematology Division, Department of Salute Della Donna e Del Bambino (SDB), University of Padua, 35128 Padua, Italy; luca.trentin78@gmail.com; 8 Medical Genetics Unit, AOU Città Della Salute e Della Scienza, 10126 Turin, Italy

**Keywords:** acute lymphoblastic leukemia, exome, DNA methylation, RNA sequencing, NRAS

## Abstract

The *KMT2A/AFF1* rearrangement is associated with an unfavorable prognosis in infant acute lymphocytic leukemia (ALL). Discordant ALL in monozygotic twins is uncommon and represents an attractive resource to evaluate intrauterine environment–genetic interplay in ALL. Mutational and epigenetic profiles were characterized for a discordant *KMT2A/AFF1*-rearranged infant monozygotic twin pair and their parents, and they were compared to three independent *KMT2A*/*AFF1*-positive ALL infants, in which the DNA methylation and gene expression profiles were investigated. A de novo Q61H NRAS mutation was detected in the affected twin at diagnosis and backtracked in both twins at birth. The *KMT2A/AFF1* rearrangement was absent at birth in both twins. Genetic analyses conducted at birth gave more insights into the timing of the mutation hit. We identified correlations between DNA methylation and gene expression changes for 32 genes in the three independent affected versus remitted patients. The strongest correlations were observed for the *RAB32*, *PDK4*, *CXCL3*, *RANBP17*, and *MACROD2* genes. This epigenetic signature could be a putative target for the development of novel epigenetic-based therapies and could help in explaining the molecular mechanisms characterizing ALL infants with *KMT2A/AFF1* fusions.

## 1. Introduction

Acute lymphoblastic leukemia (ALL) represents 25% of all diagnosed tumors among 0–14-year-old patients [1]. Leukemia in children less than one year old (infants) represents about 3% of all pediatric ALL and most frequently develops a B-cell ALL form, which is characterized by an uncontrolled proliferation of immature B-cell precursors in the peripheral blood (PB) and bone marrow [2].

The triggering genetic alteration underlying leukemogenesis is a chromosomal translocation occurring at the *KMT2A* (formerly *MLL*) gene locus (11q23), with the *KMT2A/AFF1* fusion being the most frequent cytogenetic abnormality in infant B-cell ALL. For children in the first year of life carrying this translocation, leukemia can arise as an aggressive disease with an unfavorable prognosis. It is still under debate whether *KMT2A* fusions alone are sufficient to cause clinically overt leukemia [2].

The *KMT2A* gene encodes a DNA-binding protein that methylates histone H3 and positively regulates expression of the target genes. A rigorous regulation of DNA methylation and histone modifications are essential for proper hematopoietic cell development. Genome-wide DNA methylation alterations have been described in several studies as able to distinguish ALL samples recruited at diagnosis from matched remission samples and to discriminate B-ALL from T-ALL, as well as B-ALL subtypes. In particular, infants with *KMT2A* rearrangements exhibit characteristic DNA methylation patterns, depending on the translocation partner, although they all display DNA hypermethylation when compared with normal samples [3]. Considering the even more established role of epigenetics in leukemia subtype classification, prognosis, and progression, DNA methylation signatures have also been suggested as a complementary method for ALL diagnosis in undefined ALL patient groups [4].

Monozygotic twins discordant for leukemia represent a precious opportunity to evaluate the interplay between the intrauterine environment and genetic factors, as they are matched for genotype, age, gender, paternal age, and exposure to several common environmental factors [5]. In those children who share genetic identities and the prenatal environment, the concordance rate of ALL occurring during the first months of life usually reaches 100% [6]. In the present study, we characterized for the first time the mutational and DNA methylation profiles of a case of monozygotic twins discordant for an infant B-ALL with the *KMT2A/AFF1* translocation.

## 2. Results

### 2.1. KMT2A/AFF1 Discordancy among Monozygotic Twin Samples

**Figure 1:**
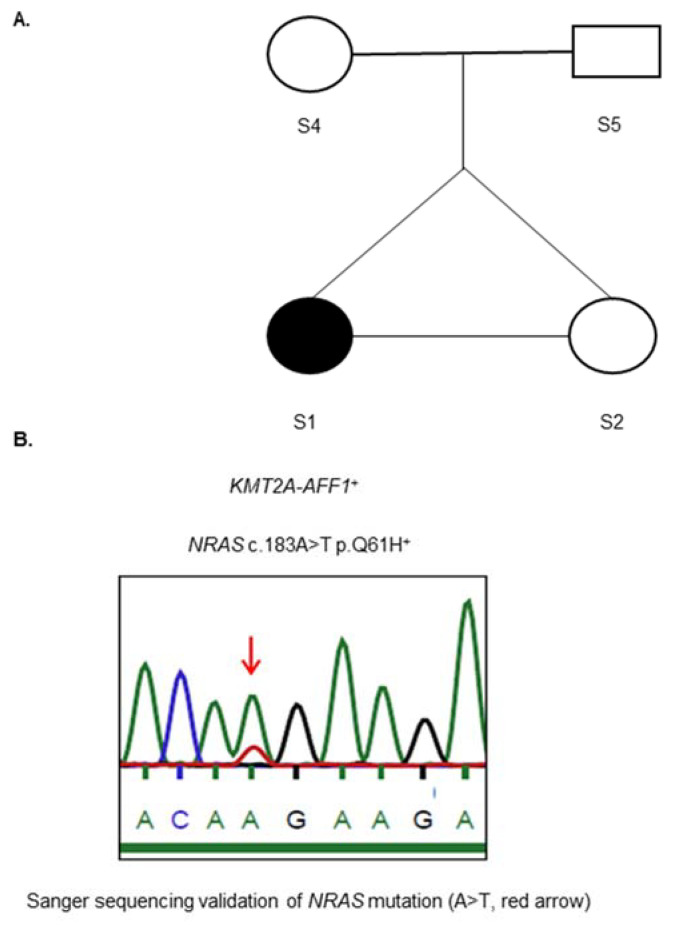
(**A**) Twins’ family tree. (**B**) Genetic alterations in the proband at diagnosis.

At the age of 5 months, the twin S1 (Figure 1A) was diagnosed as having pro-B-ALL, with t(4;11) involving *KMT2A* intron 12 (chromosome 11q23) and *AFF1* intron 5 (chromosome 4q23) (Supplementary Figure S1). Twin S1 had the clonal *IGH* VH6-JH3 rearrangement, and no chromosomal translocation involving the *KMT2A* and *AFF1* genes was revealed in the healthy twin sister (S2). Of note, no amplification signal for either *KMT2A/AFF1* or *IGH* VH6-JH3 was detected at birth in the siblings (affected twin: 0/30,000 cells with *KMT2A/AFF1* and 0/18,000 cells with *IGH* VH6-JH3; Healthy twin: 0/39,000 cells with *KMT2A/AFF1* and 0/9000 cells with *IGH* VH6-JH3).

### 2.2. Exome Sequencing on Monozygotic Twins Discordant for ALL

To identify ALL-linked de novo somatic mutations, the siblings and parents were subjected to WES. No high-confidence de novo somatic indel changes were detected in the proband. Among the de novo somatic variants of the proband with global allele frequencies lower than 1%, we detected the missense heterozygous mutation *NRAS* c.183A > T p.Q61H (variant allele frequency = 26.7%) as being absent in all the healthy samples and confirmed this using Sanger sequencing (Supplementary Table S1) (Figure 1B).

In order to determine whether the *NRAS* mutation was already present at birth or acquired afterward, neonatal blood spots of the twins were scrutinized to search for NRAS p.Q61H mutation by ddPCR, and 0.13 copies/µL and 0.17 copies/µL of a mutant allele were detected in both the proband and the healthy twin sister, respectively.

### 2.3. Differential DNA Methylation and Gene Expression

In all five family samples (S1–S5), a panel of 393,797 CpG was obtained.

In order to specifically identify ALL-related DNA methylation changes in the affected child (S1), we focused our analysis on 8120 CpGs, satisfying the following criteria: ∆β-values of at least 30% between the proband (S1) and both her healthy sister (S2) and the remitted sample (S3) and ∆β-values ≤10% among the healthy samples (S2 and S3). Most of the CpGs (6599) were consistently hypermethylated in the proband at diagnosis compared with the healthy samples (∆β-values between 30% and 82%). Among the 8120 CpG sites identified within the twin samples, 5139 were also differentially methylated with similar effects (i.e., *t*-test mean differences of at least 30%) and the same directions among the three diagnosis–remission-matched samples (couples A1_d_–A1_r_, A2_d_–A2_r_, and A3_d_–A3_r_) which were derived from three independent (and unrelated) B-ALL infants carrying the *KMT2A/AFF1* chromosomal rearrangement (Supplementary Table S2). The majority of these CpGs (4396) were hypermethylated at diagnosis (30–85% difference) in accordance with what was observed within the twin samples.

In total, the 5139 differentially methylated CpG sites were annotated to 1904 gene regions. The top 20 GSEA results are listed in Supplementary Table S3.

**Figure 2:**
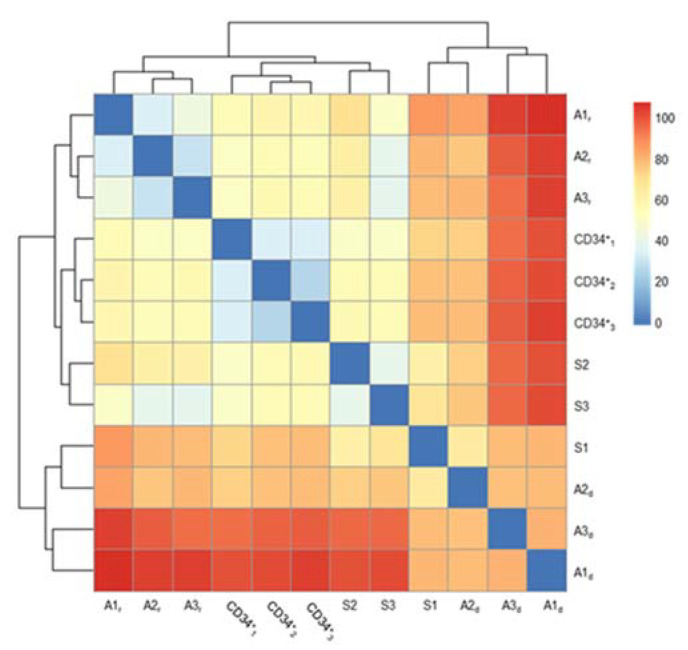
Unsupervised cluster heatmap of the Euclidean distances (0–100) among the twin samples (S1, S2, and S3), the three diagnosis–remission-paired samples (couples Ai_d_–Ai_r_, i = 1, 2, 3), and the CD34_i_^+^ (i = 1, 2, 3) cells based on their DNA methylation values for 398,224 CpG loci.

All samples collected at diagnosis (S1, A1_d_, A2_d_, and A3_d_) consisted predominantly of immature B-cells, whereas the remission samples (S3, A1_r_, A2_r_, and A3_r_) included a mixture of different mature blood cell populations. To take this difference into account, we subjected the entire methylation dataset shared by the twins (S1, S2, and S3), the three diagnosis–remission-paired samples (couples A1_d_–A1_r_, A2_d_–A2_r_, and A3_d_–A3_r_) and the CD34^+^ hematopoietic stem cells (398,224 CpG sites) to unsupervised clustering analysis. As shown in Figure 2, the first separation clearly divided the affected samples from the healthy samples.

**Figure 3:**
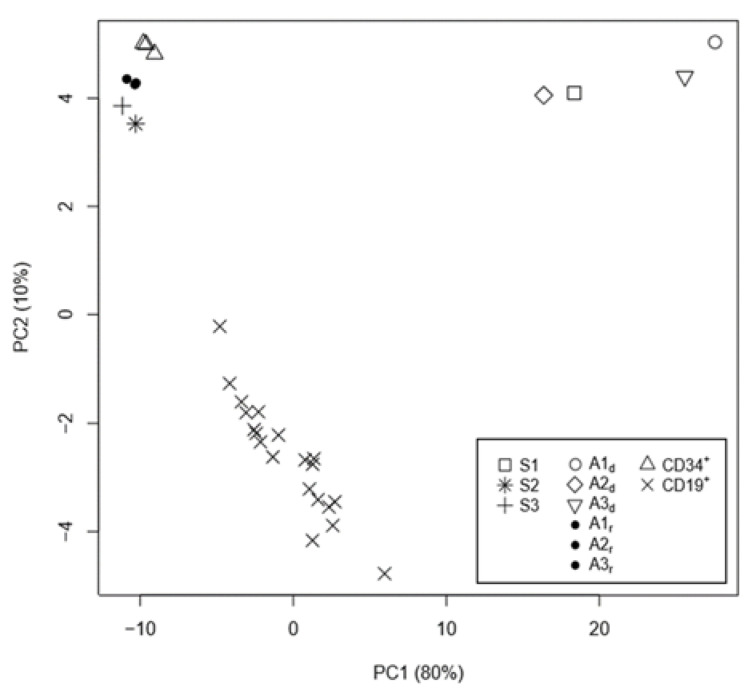
Principal component analysis (PCA) of the DNA methylation values for the “top” 5139 CpG loci among the twin samples (S1, S2, and S3), the three diagnosis–remission-paired samples (couples Ai_d_–Ai_r_, i = 1, 2, 3), and the CD34^+^ and CD19^+^ cells.

To verify that the “top” 5139 differentially methylated CpG sites corresponded to a signature characteristic of B-ALL, independent from the different proportion of blood cells in the samples, we compared the CpG site methylation levels among the leukemic samples (S1, A1_d_, A2_d_, and A3_d_) and non-leukemic samples (S2, S3, A1_r_, A2_r_, and A3_r_) and the non-leukemic CD19^+^ B cells and CD34^+^ cells isolated from the adult donors and cord blood samples, respectively. This set of non-leukemic reference cells represented mature B-cells (CD19^+^) and multipotent progenitor cells (CD34^+^). The “targeted” PCA (Figure 3) revealed a cluster of B-ALL-affected individuals distinct from the non-leukemic individuals and reference samples. PC2 separated the CD19^+^ cells from all the other samples. We imputed this result to the different ages between the CD19^+^ cell donors (adults) versus the infant samples.

Whole transcriptome profiles were determined for the four diagnosis–remission samples through mRNA sequencing. Unsupervised hierarchical clustering revealed expression profiles distinguishing the diagnosis samples from the remission samples (Supplementary Figure S2). Specifically, we identified 4376 differentially expressed genes in the diagnosis versus remission samples (1.44 < |Log2FoldChange (FC)| < 12.34; 6.03 × 10^−42^ < PFDR < 0.05; Supplementary Table S4). The downregulated (2536) and upregulated (1840) genes at diagnosis included genes involved in the development or functioning of the immune system, response to stimuli, and characterization of the immune cell subtypes. The top 20 significant overlaps computed for both the down- and upregulated genes are provided in Supplementary Table S5A,B.

Among the 4376 genes significantly up- and downregulated at diagnosis, 363 (8.3%) overlapped those harboring the 5139 differentially methylated CpG sites in both of the twin samples and in the three paired diagnosis–remission samples. We correlated the CpG methylation changes and expression levels. Thirty-two out of 363 genes harbored at least 3 CpGs (134 CpG sites in total; Supplementary Table S6), with rho ≥ |0.7|. Notably, in the healthy parents (S4 and S5), the methylation profiles of these 134 CpG sites were comparable to those of an internal control group composed of healthy adult subjects (data not shown), which excluded possible deregulated patterns potentially transmitted from parents to their daughters.

**Figure 4:**
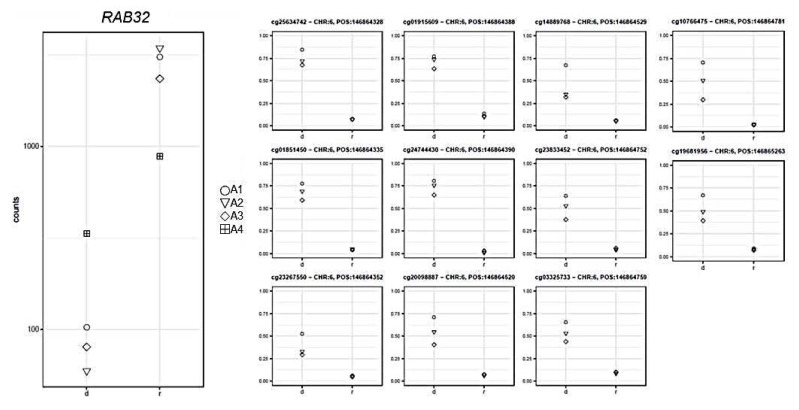
Gene expression (left panel) and corresponding methylation level (*y*-axis, right panel) comparison between samples at diagnosis (d) and remission (r) for the *RAB32* gene.

GSEA analysis performed on the 32 genes whose expression levels correlated with the DNA methylation profiles showed significant overrepresentation of genes upregulated in embryonic stem cells from *TCEB3* knock-out mice, cell–cell junctions, and cell movements (FDR *q*-value < 9.36 × 10^−3^; Supplementary Table S7). The differentially methylated CpG sites corresponding to these 32 genes were all hypermethylated at diagnosis, and 65% of them were within the gene promoter. Negative correlations were observed between methylation and gene expression for all CpGs except for those within the *MACROD2* gene and one CpG in the *PLEKHG5* 3′UTR. The expression levels of five genes showed the strongest significance between the affected and healthy subjects (PFDR < 10^−6^) as well as a correlation higher than 80% with corresponding DNA methylation changes. In particular, the methylation levels of 11 CpG sites located within 1500bp or 200bp upstream of the transcription starting site (TSS) of the *RAB32* gene were inversely correlated with their expression levels (log2FC = −4.42; PFDR = 4.08 × 10^−8^; −0.97 ≤ rho ≤ −0.84; Pcorr ≤ 0.04; Figure 4).

**Figure 5:**
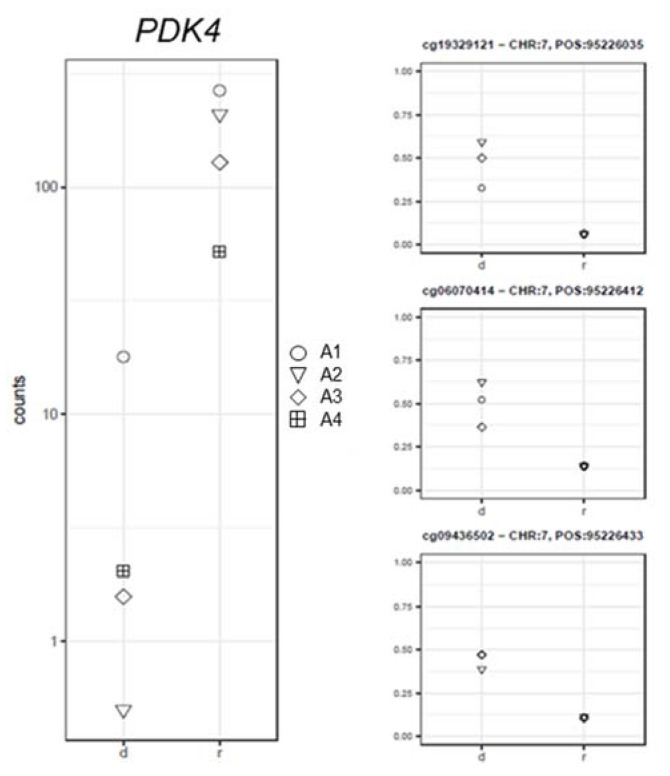
Gene expression (left panel) and corresponding methylation level (right panel) comparison between the samples at diagnosis (d) and remission (r) for the *PDK4* gene.

An inverse correlation was also observed between the β-values of three CpGs within the TSS of the *PDK4* gene and mRNA levels (log2FC = −6.07; PFDR = 1.26 × 10^−8^; −0.95 ≤ rho ≤ −0.89; Pcorr ≤ 0.02; Figure 5).

**Figure 6:**
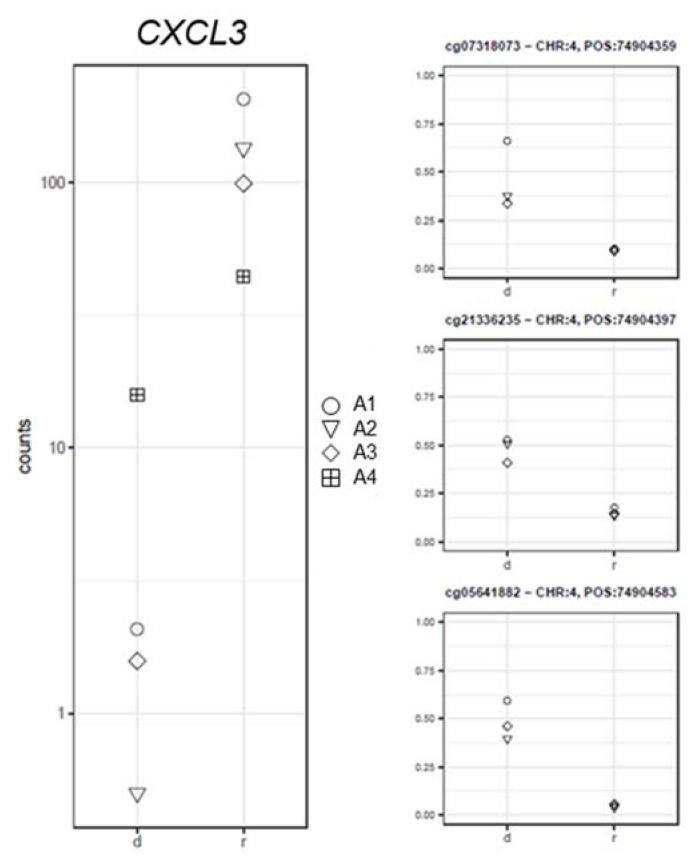
Gene expression (left panel) and corresponding methylation level (right panel) comparison between the samples at diagnosis (d) and remission (r) for the *CXCL3* gene.

**Figure 7:**
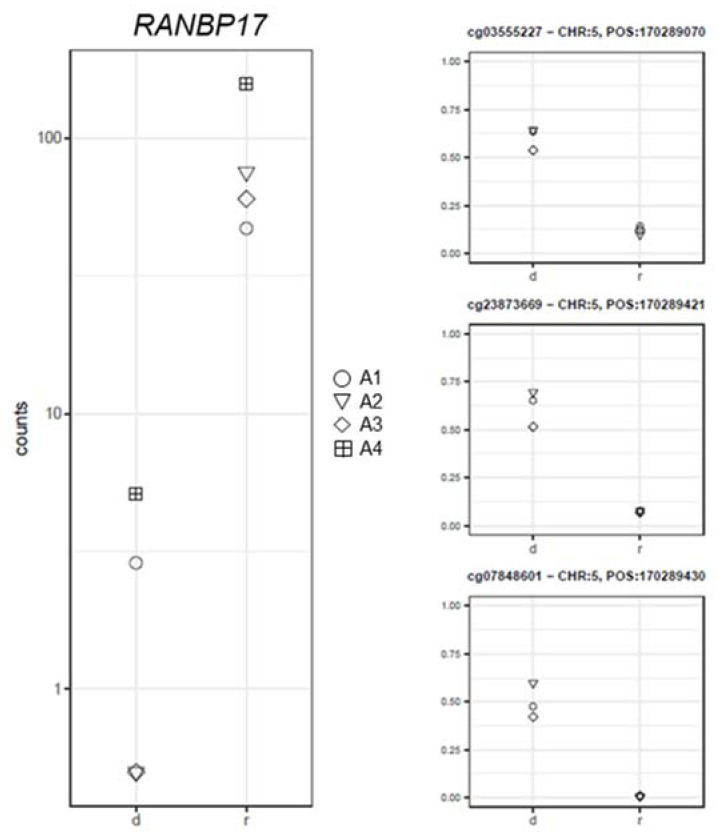
Gene expression (left panel) and corresponding methylation level (right panel) comparison between the samples at diagnosis (d) and remission (r) for the *RANBP17* gene.

Moreover, the *CXCL3* and *RANBP17* gene expression values negatively correlated with the corresponding CpGs methylation levels (*CXCL3*: log2FC = −6.09; PFDR = 1.03 × 10^−7^; −0.95 ≤ rho ≤ −0.85; Pcorr ≤ 0.04 (Figure 6) and *RANBP17*: log2FC = −6.01; PFDR = 5.38 × 10^−8^; −0.93 ≤ rho ≤ −0.91; Pcorr ≤ 0.02; Figure 7).

**Figure 8:**
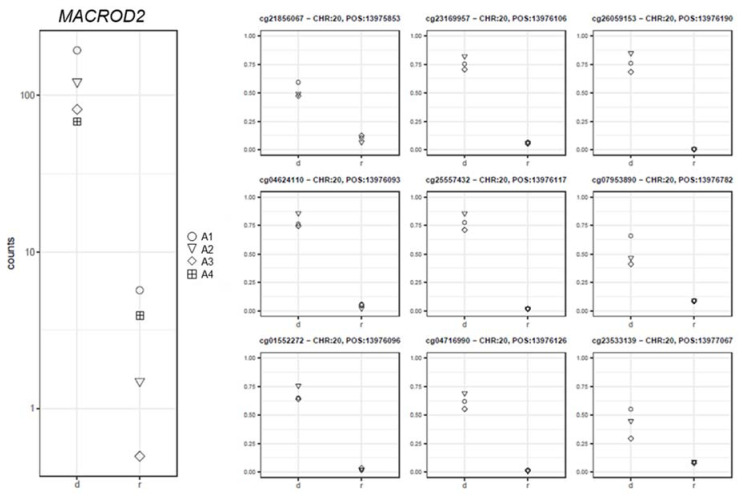
Gene expression (left panel) and corresponding methylation level (right panel) comparison between the samples at diagnosis (d) and remission (r) for the *MACROD2* gene.

Conversely, the *MACROD2* expression levels decreased at remission and showed a positive association with the methylation levels of nine CpGs (log2FC = 5.83; PFDR = 3.18 × 10^−9^; 0.86 ≤ rho ≤ 0.99; Pcorr ≤ 0.03; Figure 8).

## 3. Discussion

In the current study, to identify new potential molecular mechanisms characterizing *KMT2A/AFF1*^+^ B-ALL, we investigated, for the first time, the case of monozygotic twins with the discordant diagnosis of *KMT2A/AFF1* leukemia by exploiting different “-omics” approaches. Exome sequencing showed only the de novo NRAS Q61H mutation. Whole-genome sequencing has previously revealed a mutational landscape characterized by few non-silent alterations in ALL infants with rearrangements of the *KMT2A* gene [7,8]. This is consistent with the very short latency period between *KMT2A* rearrangement onset (often occurring in utero) and the clinically overt leukemia [9,10]. The NRAS Q61H mutant allele frequency well below 50% in the proband confirms the subclonal nature of the mutation [7]. A high frequency of RAS mutations has been reported in infant patients, supporting the hypothesis that RAS activation may be responsible for the shortened latency [11]. Moreover, *NRAS* mutations seem to contribute to the adverse prognosis typical of *KMT2A*-rearranged infant patients [12,13,14,15].

In our study, the *NRAS* mutation was also backtracked in the siblings’ neonatal blood spots, suggesting that the mutation event arose during the prenatal period. In contrast, no pre-leukemic cells with the *KMT2A/AFF1* rearrangement were detected in either twins at birth. These results offer two hypothetical scenarios: (1) a post-natal chromosomal rearrangement event, which would indicate that the *NRAS* subclonal mutation preceded the chromosomal aberration, a phenomenon recently described in an infant with acute myelomonocytic leukemia [16], or (2) undetectable rearranged blasts could have been present in both the neonatal twins. Considering the fact that most ALL infants could have less than one pre-leukemic blast of over 104 nucleated cells at birth [17], and we did not even observe the patient-specific clonal *IGH* VH6-JH3 rearrangement, we could not exclude the presence of a silent pre-leukemic clone. We could speculate the existence of a clone with the *KMT2A/AFF1* fusion, which competes with the *NRAS*-mutated clone and initiates the leukemic process, and a minor, which may accelerate the development of the overt leukemia.

The discordancy for leukemia in monozygotic twins is very rare [6,18,19,20]. Placental anastomoses together with the early onset of the disease support a prenatal origin of leukemia, with a concordance rate among identical twins reaching 100% [6]. Three hypotheses could explain the discordancy for ALL: (1) The pre-leukemic clones remain in a dormant state in the healthy child, as it was shown that, occasionally, pre-leukemic clones can last up to 14 years [21]. (2) The clearance of the pre-leukemic clones happened within the healthy infant. This speculation is based on the adrenal hypothesis that proposes an anti-leukemic effect as a consequence of cortisol secretion occurring during an early infectious process [22]. (3) The MLL rearrangement was post-natal in the ALL twin only [16].

Aside from genetic alterations, we reported B-ALL-associated DNA hypermethylation in the *KMT2A/AFF1*-positive twin infant at diagnosis, which reverted after chemotherapy treatment. DNA methylation changes were replicated in three independent diagnosis–remission-matched samples carrying the *KMT2A/AFF1* rearrangement.

GSEA performed on the diagnosis vs. remission differentially methylated genes highlighted a significant enrichment for target genes of polycomb repressive complex 2, a histone methyltransferase associated with gene repression which plays a fundamental role in the fine regulation of pluripotent stem cells [23]. Among significantly different expressed genes, crucial leukemogenic genes known to be targets of the *KMT2A/AFF1* fusion protein (for example, *HOXA*s and *MEIS1* genes) were upregulated at diagnosis, as expected [24]. We also reported DNA methylation changes that mostly translated into significantly downregulated genes related to *TCEB3* expression (Supplementary Table S7). The protein product of *TCEB3*, Elongin A, forms a stable complex with Elongins B and C, which are required to maintain low expression levels of PCR2-repressed genomic targets [25,26]. We observed the strongest correlations between methylation changes and corresponding gene expression for the *RAB32*, *PDK4*, *RANBP17*, *CXCL3*, and *MACROD2* genes. Rab small GTPases are a member of the RAS oncogene superfamily [27]. Aberrant DNA methylation of *RAB32* was already described in a heterogeneous group of B-ALL pediatric patients harboring different chromosomal rearrangements, including *KMT2A/AFF1*. However, in comparing leukemic samples with non-leukemic samples, *RAB32* gene expression and methylation were not significantly correlated, probably due to the presence of mixed B-ALL subtypes [28]. Higher *RAB32* mRNA expression was also described in good-prognosis pediatric patients affected by AML compared with those with poor prognosis [29] and in adult AML patients versus healthy volunteers [30]. Therefore, lower *RAB32* gene expression seems to be associated with a malignant phenotype.

*PDK4* hypermethylation has been reported for pediatric B-ALL patients with the *ETV6-RUNX1* translocation [31]. Bohl and colleagues found an association between *PDK4* expression and the lack of a response to the hypomethylating agent decitabine in AML patients. This gene is a poor prognostic marker and has been associated with EVI1- and FLT3-ITD-mediated signaling [32]. Disruption of the *RanBP17/Hox11L2* region was described in ALL children [33].

The chemokine *CXCL3* was found to be epigenetically repressed in B-cell ALL [28]. Consistently, this gene was downregulated in our patients at diagnosis. Chemokine signaling in ALL may have a role in the localization of leukemic blasts in specific niches, and it may confer resistance to chemotherapy [34].

In the samples at diagnosis, *MACROD2* expression was considerably higher than in the remission samples. Translocations affecting the *MACROD2* gene were reported in a recurrent hyperdiploid ALL pediatric patient at relapse, characterized by several chromothriptic events [35,36]. A number of studies suggest a potential role of *MACROD2* in cancer; however, it is not clear whether high or low *MACROD2* expression levels contribute to tumorigenesis [37]. We reported the lack of an inverse correlation between *MACROD2* expression levels and the promoter methylation, which may indicate a different mechanism of gene expression regulation, such as histone modifications in the promoter or the involvement of different isoforms. In summary, these five top-ranked genes were reported here for the first time as deregulated genes in *KMT2A/AFF1* infants.

Among the other genes with strong correlations between DNA methylation and gene expression (Supplementary Table S6), aberrant DNA methylation and downregulation of *FKBP9*, *CA8*, *MSC*, *TRPC6*, *ZNF492*, *ZNF229*, and *ZNF471* genes in leukemia patients are reported here for the first time.

New potential therapeutic targets are necessary, particularly for infants who respond poorly to conventional chemotherapy, and allogeneic hematopoietic stem cell transplantation with an HLA-matched donor is recommended [38]. Given the reversible nature of methylation changes, they represent attractive therapeutic targets. DNA methylation profiling could be important to monitor the efficacy of these novel therapies. Moreover, the accurate assignment of patients to specific risk groups is currently a difficult and expensive process, requiring immunophenotyping, cytogenetics, and molecular diagnostics [39,40]. To conclude, the signature consisting of 32 genes whose methylation levels were reported here to be strongly and significantly correlated with expression changes could be a putative target for the development of novel epigenetic-based therapies and are worthy of further investigations to better understand the molecular mechanisms underlining the dismal prognosis of ALL infants with *KMT2A/AFF1* fusions.

## 4. Materials and Methods

### 4.1. Cases and Biological Samples

Monochorionic twin sisters were born by cesarean section at 35 weeks of gestation, conceived after monochorionic in vitro fertilization. At 5 months of age, one child was diagnosed as pro-B ALL positive for the translocation *KMT2A/AFF1*, with 75% of blasts at the cytofluorimetric analysis (CD10^−^, CD19^+^) and 80% of blasts at the morphological analysis. The patient was treated as a high-risk infant ALL according to the INTERFANT-06 protocol (EudraCT Number: 2005-004599-19; ClinicalTrials.gov Identifier: NCT00550992) in AIEOP (Associazione Italiana Ematologia Oncologia Pediatrica) centers in Italy. After induction chemotherapy, she achieved complete remission and then received cord blood allogenic hematopoietic stem cell transplantation from an unrelated donor.

Mononuclear cells were collected from the affected child at diagnosis (S1) and at complete remission prior to allogeneic hematopoietic cell transplantation (S3), as well as from her healthy monozygotic twin sister (S2) (Figure 1A). PB was also collected from their healthy parents (S4, S5) (Figure 1A). DNA samples were extracted using a QIAamp DNA Blood Kit (Qiagen, Hilden, Germany).

PB from the twin sisters was drawn during the first hours post-partum and spotted onto Guthrie cards. Neonatal DNA was extracted using a QIAamp DNA Mini Kit (Qiagen, Hilden, Germany) from a quarter of the Guthrie cards. Details on breakpoint sequencing and searching for rearrangements in the twin sisters at birth and at diagnosis are provided in Appendix A.1.

To perform *NRAS* mutation analysis in the twin sisters’ neonatal spots, DNA was extracted from half of the Guthrie cards using the QIAamp DNA Investigator FTA and Guthrie cards kit (Qiagen, Hilden, Germany). DNA was quantified using the Quant-iT High-Sensitivity dsDNA Assay Kit (Thermo Fisher Scientific, Waltham, MA, USA).

In addition, we recruited four unrelated samples from infant patients with clinical characteristics in line with the twin specimens. The samples (i.e., A1_d_–A1_r_, A2_d_–A2_r_, A3_d_–A3_r_, and A4_d_–A4_r_) were collected at the time of diagnosis (_d_) and remission (_r_); DNA (A1_d_–A1_r_, A2_d_–A2_r_, and A3_d_–A3_r,_) or RNA (A1_d_–A1_r_, A2_d_–A2_r_, A3_d_–A3_r_, and A4_d_–A4_r_) were isolated from the total bone marrow or PB (Supplementary Table S8).

Informed consent was obtained from the parents. The present study was carried out in accordance with the ethical principles of the Declaration of Helsinki.

### 4.2. Whole-Exome Sequencing

Whole-exome sequencing (WES) was conducted on five samples (S1–S5) as detailed in Appendix A.1.

### 4.3. Sanger Sequencing

*NRAS* exon 3 was subjected to Sanger sequencing at the Eurofins Genomics facilities (Ebersberg, Germany) to confirm the presence of the p.Q61H mutation identified by WES in the proband sample at diagnosis as well as its absence in her healthy twin sister and in their parents.

### 4.4. NRAS Mutation Analysis in the Twin Sisters’ Neonatal Spots

The presence of the *NRAS* c.183A > T p.Q61H mutation was assessed using the QuantStudio 3D Digital PCR instrument and chips (Thermo Fisher Scientific, Waltham, MA, USA). Absolute quantification of the target sequence was obtained through 20,000 simultaneous PCR reactions. Details are provided in Appendix A.1. The results are reported as copies per microliters (copies/µL) calculated from the fluorescence signal generated by the amplification of the mutant allele targeted by the FAM dye-labeled probe and the wild-type allele targeted with the VIC dye-labeled probe.

### 4.5. DNA Methylation and mRNA Expression Analysis

Samples S1–S5 and A1_d_–A1_r_, A2_d_–A2_r_, and A3_d_–A3_r_ underwent DNA methylation analysis using the HumanMethylation450 BeadChip (Illumina, San Diego, CA, USA). The total RNA of four B-ALL patients at diagnosis (A1_d_, A2_d_, A3_d_, and A4_d_) and the same four patients at remission (A1_r_, A2_r_, A3_r_, and A4_r_) were also processed using the TruSeq RNA Sample Prep V2 (Illumina, San Diego, CA, USA) to perform whole-transcriptome analysis. Additional information and statistical analysis are presented in Appendix A.1.

### 4.6. Statistical Analysis

In order to identify the most differentially methylated CpG sites among the proband, the remitted proband, and the healthy monozygotic sister, a threshold of at least 30% methylation difference was set (i.e., | β-value_proband_ − β-value_remission_| ≥ 0.3 and | β-value_proband_ β-value_healthy sister_| ≥ 0.3). Similarly, to ensure that the remaining sites were not differentially methylated between the healthy twin and the remitted proband, a threshold of at most 10% methylation difference was fixed. We named the ∆β-value the difference in the methylation level of the same CpG site between two different individuals. The resulting CpG sites were then intersected with the differentially methylated ones in the three B-ALL diagnosis–remission-matched samples, previously obtained through a paired *t*-test (|effect size| > 30%).

Principal component analysis (PCA), together with cluster heatmaps, was conducted considering the S1, S2, and S3 samples together with independent B-ALL diagnosis–remission-matched samples (A1_d_–A1_r_, A2_d_–A2_r_, and A3_d_–A3_r_) and methylation profiles from CD34^+^ hematopoietic stem cells isolated from fresh umbilical cord blood derived from three healthy blood donors (GEO Accession Number: GSE40799). CD19^+^ cells from 20 healthy adult blood donors were also used as non-leukemic reference cells (GEO Accession Number: GSE49031).

For multiple comparison tests (Bioconductor package DeSeq2) performed on whole-gene expression data generated for four acute leukemia diagnosis–remission couples (A1_d_–A1_r_, A2_d_–A2_r_, A3_d_–A3_r_, and A4_d_–A4_r_), a Benjamini–Hochberg false discovery rate (FDR) *p*-value (P_FDR_) lower than 0.05 was considered statistically significant.

The “top” CpG sites (i.e., those identified within the twin samples and the three diagnosis–remission-matched samples) were mapped to genes also significantly differentially expressed and underwent subsequent analyses of the Pearson correlation between their methylation level and the expression variation of the corresponding gene. This possible relationship was assessed in the three B-ALL diagnosis–remission couples, because both DNA and RNA were available for them.

All the analyses were performed with the open source R (R 3.1.0) package.

Gene enrichment analysis was performed using the gene set enrichment analysis computational method (GSEA; Broad Institute; http://software.broadinstitute.org/gsea (accessed on 31 December 2019)) to search for a statistically significant set of genes. The annotated gene sets are collected in the Molecular Signatures Database (MSigDB) v6.2 [41]. Enrichments with *p*-values < 0.05 after correction for multiple comparisons by FDR were considered statistically significant.

## Data Availability

Data are contained within the article or Supplementary Materials.

## Supplementary Materials

The following are available online at https://www.mdpi.com/article/10.3390/ijms22189740/s1. Figure S1: Breakpoint sequence for *KMT2A*/AFF1 rearrangement. Figure S2: Unsupervised clustering analysis of the Euclidean distances of gene expression data (30,864 genes) in the four diagnosis–remission-paired samples (couples Ai_d_–Ai_r_, i = 1, 2, 3, 4). Table S1: Sample S1. De novo single-nucleotide variants within gene regions and with a population frequency <1%. Table S2: CpG sites with at least 30% methylation differences between the twin samples (S1 and S2) and between three diagnosis–remission-matched samples (A1_d_–A1_r_, A2_d_–A2_r_, and A3_d_–A3_r_). Table S3: Gene set enrichment analysis. The top 20 overlap with the false discovery rate (FDR) (*q*-value < 0.05) computed for 1904 genes. Table S4: Genes with significantly different expression levels in the diagnosis (A1_d_, A2_d_, A3_d_, and A4_d_) versus remission (A1_r_, A2_r_, A3_r_, and A4_r_) samples. Table S5-A: Gene set enrichment analysis. The top 20 overlap with the FDR (*q*-value < 0.05) computed for the 2536 downregulated genes. Table S5-B: Gene set enrichment analysis. The top 20 overlap with the FDR (*q*-value < 0.05) computed for the 1840 upregulated genes. Table S6: Correlation between DNA methylation and gene expression levels. Table S7: Gene set enrichment analysis performed on 32 genes with significant correlations between DNA methylation and gene expression. Table S8: Clinical characteristics of the four unrelated leukemia patients.

## Appendix A

### Appendix A.1. Supplementary Methods A1

#### Appendix A.1.1. Breakpoint Sequencing, Minimal Disease Monitoring, and Rearrangement Testing

The *KMT2A/AFF1* breakpoint was sequenced at the Diagnostic Center of Acute Leukemia (DCAL) at Goethe University Frankfurt, Germany. Based on the genomic breakpoint, a DNA-based quantitative PCR (qPCR) assay was set up to monitor the genomic breakpoint with a sensitivity of 1 × 10^−4^. The clonal immunoglobulin and T-cell receptor somatic gene rearrangements were identified at diagnosis [42] to be used for minimal residual disease monitoring. The qPCR for the *IGH* VH6-JH3 rearrangement showed a sensitivity of 1 × 10^−5^.

The neonatal DNA quantity was assessed by qPCR amplification of the albumin gene, and the qPCR was used to evaluate the presence of both the *KMT2A/AFF1* and the patient-specific clonal *IGH* VH6-JH3 rearrangement identified at diagnosis. For the affected twin (S1), 10 replicates were performed to test for the *KMT2A/AFF1* translocation, and 6 were performed for *IGH* VH6-JH3. For the twin sister (S2), 13 replicates were carried out for the *KMT2A/AFF1* arrangements and 3 for the *IGH* VH6-JH3 rearrangements. The amount of DNA for each replicate was retrieved from about 3000 cells.

#### Appendix A.1.2. Whole-Exome Sequencing

Whole-exome sequencing (WES) was conducted on five samples (S1–S5) using the SureSelect Human All Exon V5+UTRs (74.6 Mb) enrichment panel and the SureSelectQXT protocol (Agilent Technologies, Santa Clara, CA, USA). Exome-enriched libraries were sequenced on the NextSeq500 platform (Illumina, San Diego, CA, USA). The reads were aligned to the human reference genome hg19 using a Burrows–Wheeler aligner (BWA-0.7.10) [43]. Variant calling and hard filtering were performed using Genome Analysis ToolKit (GATK)-HaplotypeCaller v3.2-2 and GATK-VariantFiltration v3.2-2 [44]. Visual exploration of the sequencing data was conducted using the Integrated Genomics Viewer tool (IGV; Broad Institute, http://software.broadinstitute.org/software/igv/ (accessed on 31 December 2019)). The variant frequencies were assessed using the 1000 Genomes (http://www.1000genomes.org/ (accessed on 31 December 2019)) and ExAc databases (http://exac.broadinstitute.org/ (accessed on 31 December 2019)). Variant annotation and prioritization were performed using different tools: VariantStudio software (Illumina, San Diego, CA, USA), Alamut (Interactive Biosoftware, Rouen, France), Mutation Assessor (http://mutationassessor.org/ (accessed on 31 December 2019)), Provean (http://provean.jcvi.org/ (accessed on 31 December 2019)), and Mutation Taster (www.mutationtaster.org/ (accessed on 31 December 2019)). Variants causing amino acid changes and with deleterious or damaging predictions in at least three out of five prediction tools were selected.

#### Appendix A.1.3. NRAS Mutation Analysis in the Twin Sisters’ Neonatal Spots

The presence of the *NRAS* c.183A > T p.Q61H mutation was assessed using the QuantStudio 3D Digital PCR instrument and chips (Thermo Fisher Scientific, Waltham, MA, USA). Absolute quantification of the target sequence was obtained through 20,000 simultaneous PCR reactions.

The purchased primers and probes for the NRAS Q61H mutation analysis were the TaqMan^®^ SNP Genotyping Assay (Thermo Fisher Scientific, assay ID C_166032675_10). The digital PCR mixture (15 µL) included 7.5 µL 2 ×  QuantStudio^®^ 3D Digital PCR Master Mix v2 (Thermo Fisher Scientific, Waltham, MA, USA), 0.75 µL TaqMan^®^ SNP Genotyping Assay, 6.75 µL DNA (10 ng), and distilled water. Using the QuantStudio 3D Digital PCR Chip Loader, the mixture was loaded on a chip of the QuantStudio 3D Digital PCR 20K Chip Kit v2. The chip was amplified using the ProFlex 2 × flat PCR system (Thermo Fisher Scientific, Waltham, MA, USA) under the following conditions: 96 °C for 10 min, followed by 40 amplification cycles (2 min at 60 °C, 30 s at 98 °C) and 2 min at 60 °C, followed by a cooling step at 10 °C. After amplification, the chip was read using QuantStudio 3D and analyzed with QuantStudio 3D Analysis Suite cloud software. The results were reported as copies per microliters (copies/µL), calculated from the fluorescence signal generated by the amplification of the mutant allele targeted by the FAM dye-labeled probe and the wild-type allele targeted with the VIC dye-labeled probe.

#### Appendix A.1.4. DNA Methylation Analysis

Samples S1–S5 and A1_d_–A1_r_, A2_d_–A2_r_, and A3_d_–A3_r_ underwent DNA methylation analysis. The samples were processed in two batches.

The quality- and quantity-checked DNA (500 ng) were treated with bisulfite (EZ-96 DNA Methylation-Gold Kit, Zymo Research Corporation, Irvine, CA, USA), and the bisulfite-converted DNA was then hybridized onto the HumanMethylation450 BeadChip (Illumina, San Diego, CA, USA) [45]. The BeadChips were scanned with a HiScan SQ instrument (Illumina, San Diego, CA, USA), and the fluorescence intensities corresponding to the methylation level of each CpG were recorded.

The raw data quality check and methylation Beta (β)-value computation were previously described in [46].

Methylation β-values with a detection *p*-value ≥0.01 were excluded from the analysis, as well as CpG loci with a detection *p*-value ≥0.01 in more than 20% of the assayed samples. All the samples had a global call rate higher than 95%.

The CpG sites were annotated to gene regions according to the Infinium HumanMethylation450K Manifest.

#### Appendix A.1.5. mRNA Expression Analysis

The total RNA of four B-ALL patients at diagnosis (A1_d_, A2_d_, A3_d_, and A4_d_) and the same four patients at remission (A1_r_, A2_r_, A3_r_, and A4_r_) were extracted using TRIzol reagent (Invitrogen, Thermo Fisher Scientific, Waltham, MA, USA). RNA integrity was assessed using the Fragment Analyzer Automated CE System (Advanced Analytical, Heidelberg, Germany). At least 200 ng of samples with an RNA integrity number ≥7 was processed using the TruSeq RNA Sample Prep V2 (Illumina, San Diego, CA, USA) to prepare cDNA libraries. Whole-transcriptome single-end (1 × 75 cycles) sequencing was performed on the NextSeq500 platform (Illumina, San Diego, CA, USA). Bowtie v2.3.2 [47] was used to align the generated reads to the human genome GRCh38 and to count the number of reads mapped to each gene. The counts of reads that uniquely mapped to annotated genes were performed using the featureCounts program from the Subread package 1.5.3 [48]. The R language package Bioconductor DeSeq2 v1.18.1 was employed to perform differential expression analysis [49]. Only genes with a median of the counts greater than zero in the eight samples (couples Ai_d_–Ai_r_, i = 1, 2, 3, 4) were tested. The expression level of each mRNA was log2 transformed for the downstream analysis.
