# Supplementary material for: Genetic and Epigenetic Characterization of a Discordant KMT2A/AFF1-Rearranged Infant Monozygotic Twin Pair
Source: Int J Mol Sci. 2021 Sep 9;22(18):9740. doi: 10.3390/ijms22189740 (PMC8466096; doi:10.3390/ijms22189740)
Supplement: Supplementary file 1 [file ijms-22-09740-s001.zip › ijms-1351727-supplementary/Supplementary Figure S1.pdf]

TGGGTTCTGTATCCCTGGACTCAACCAACCTTGGATTGAATGTATCTGGGAAAAAATGAGTAGTTGCCTCTGTACTCT

ATGTGAACAGACTTTTTCTTGTCATTATTTCCTAAACAATACAGTATAACAACTATTTACATTGTATTAGGTATGATAAG

TAATCTAGAGATAATTTAAAGTATATGGTGGGCGGATCACTTGAAGCCAGGAGTTCGAGACCAGCCTGAGCCAACAT

GGTGAAACCCCATCTCTACTAAAAATACAAAAAATTAGCCAGGTGTGGTGGTGGGCACCTGTAGTCCCAGCTACTTG

GGAGGCTGAGGGAGGAAAATCGCTTGAACTTTGGAGGCAGAGGTTGCAGTGAGCCACTCCAGCCTGTGGTGCAGT

CTGTCACTCCAGCCTGGGTGACACAGTGAGACTCCATCTCAAAAAAAAAAAAAAAAAAAAACTATATGGGAGGATGT

GCATTTTGTTATATGCAAATGCTGCACCATTTTGTCTAGGGACTTGGGCATCCATG•AAGCCTTAATCTTTTAATAATT

AGATATTTTTATTTTCTTTCTAGGACTCTCAGCATGTCAGTTCTGTAACCCAAAACCAAAGTAAGTAAATTTGAAACTG

CTTATTGGATTGGAGAACAAAGCATAGCTTTTACTGTTTAACGTAAAAACATTAGTAAAAAATGCCCCAGCAGAGATG

AGGCCTTCCTATGTTGAAAGAATAGGAAAGGAGAACAAATATAAAACTATTCTTTATGTGGGATTTAAAATTGTTGAC

CAGGCGCAGTGGCTCATGCCTGTAATCCCAGCACTTTGGGAGTCCAAGGCAGTTGGATCGCTTGAGCCTAGGAGTT

GAAGACCAGCCTGGGCAACATGGTGAAACCCTGTCTCTACAAAATTAGAAAAATTAGCTGGGCTTGGAGTCATGTGC

CTGTGGTCCCAGCTACTTGGGAGGCTGAGGTGGGAGGATCACTTGAGCCTAGGAAGTAGAGGTTGCAGTGAGCCA

AGATTGTGGCACTGCACTCCAGCCTGGGAGACAGCACAGATCCTGTCTCAAACAAACAAAAACCAAAATAAGAT

KMT2A: Intron 12, chromosome 11q23
AFF1: Intron 5, chromosome 4q23

Supplementary Figure S1. Breakpoint sequence for the *KMT2A/AFF1*-rearrangement.
