# Supplementary material for: Genetic and Epigenetic Characterization of a Discordant KMT2A/AFF1-Rearranged Infant Monozygotic Twin Pair
Source: Int J Mol Sci. 2021 Sep 9;22(18):9740. doi: 10.3390/ijms22189740 (PMC8466096; doi:10.3390/ijms22189740)
Supplement: Supplementary file 1 [file ijms-22-09740-s001.zip › ijms-1351727-supplementary/Supplementary Figure S2.pptx]

## Slide 1
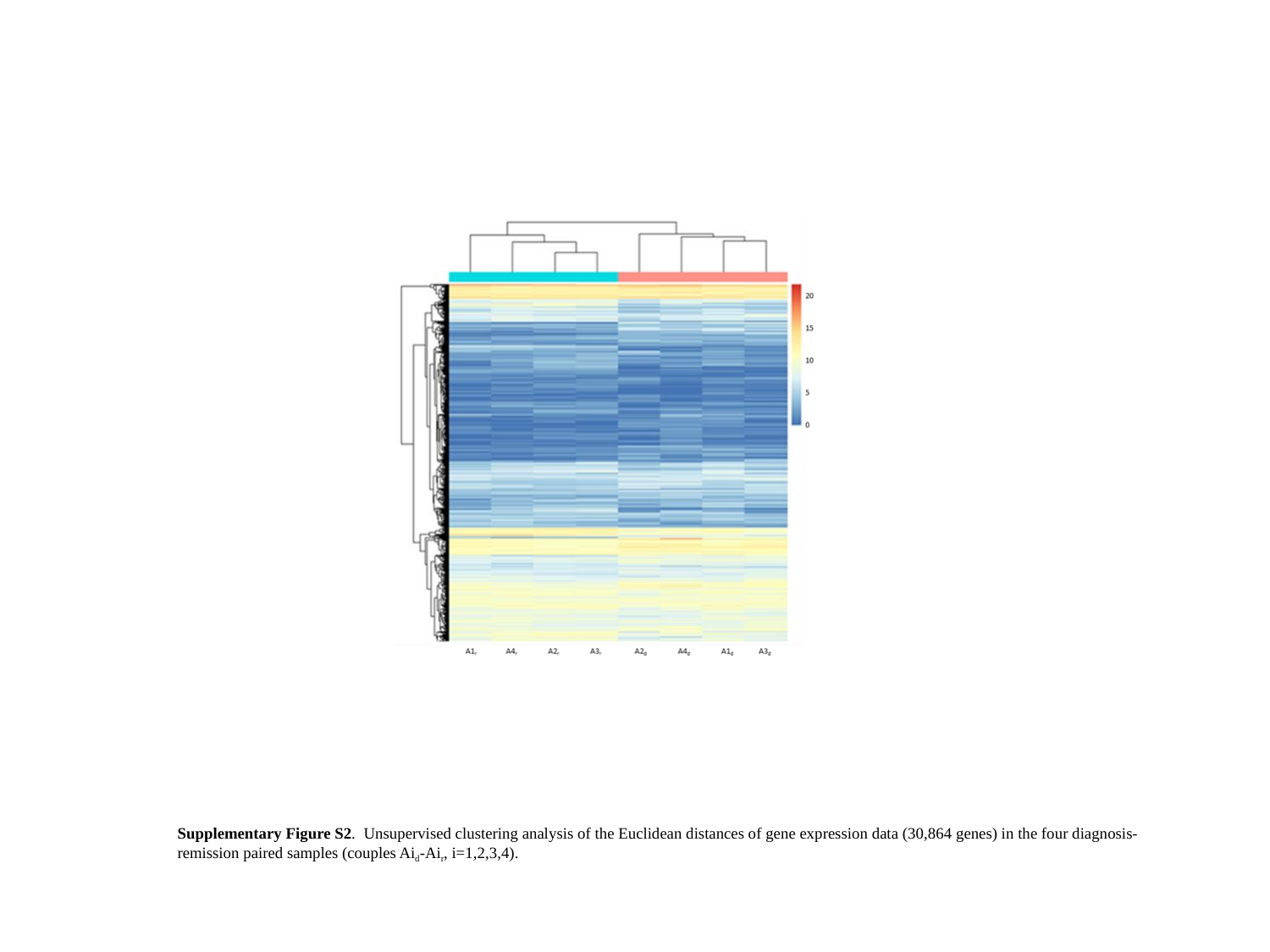

Supplementary Figure S2.  Unsupervised clustering analysis of the Euclidean distances of gene expression data (30,864 genes) in the four diagnosis-remission paired samples (couples Aid-Air, i=1,2,3,4).
